# Child Maltreatment, Peer Victimization, and Mental Health: Neurocognitive Perspectives on the Cycle of Victimization

**DOI:** 10.1177/15248380211036393

**Published:** 2021-08-06

**Authors:** Anouk Goemans, Essi Viding, Eamon McCrory

**Affiliations:** 1 Leiden University, the Netherlands; 2 University College London, United Kingdom; 3 Anna Freud National Centre for Children and Families, London, United Kingdom

**Keywords:** child maltreatment, peer victimization, mental health, stress susceptibility, stress generation, social thinning

## Abstract

Children who experience maltreatment are at increased risk of revictimization across the life span. In childhood, this risk often manifests as peer victimization. Understanding the nature of this risk, and its impact on mental health, is critical if we are to provide effective support for those children who are most vulnerable. A systematic scoping review was conducted using Google Scholar and PsycINFO. Studies on adults, psychiatric, and/or inpatient populations were excluded. Included studies concerned all forms of child maltreatment and peer victimization. We found 28 studies about the association between maltreatment experience and peer victimization as well as peer rejection. We review the evidence documenting the relation between these adverse childhood experiences and mental health. The evidence suggests that maltreatment and peer victimization have additive effects on mental health outcomes. A number of theoretical developmental frameworks that delineate putative mechanisms that might account for an association are considered. Building on prior research, we then discuss the role of recent neurocognitive findings in providing a multilevel framework for conceptualizing mental health vulnerability following maltreatment. In addition, we consider how altered neurocognitive functioning following maltreatment may shed light on why affected children are more likely to be victimized by their peers. Specifically, we consider the threat, reward, and autobiographical memory systems and their role in relation to stress generation, stress susceptibility, and social thinning. Such a mechanistic understanding is necessary if we are to reduce the likelihood of peer victimization in children exposed to maltreatment, and move to a preventative model of mental health care.

Child maltreatment is a universal problem and can have profound negative and long-lasting consequences on children’s mental health (Cyr et al., 2010; Norman et al., 2012). We do not yet have a clear mechanistic understanding of how maltreatment increases risk of mental health problems or why some children with maltreatment experience are more vulnerable to developing mental health problems than others. Any mechanistic framework needs to account for the role played by social factors that unfold *after* the maltreatment experience. Sadly, we know that children who have experienced childhood maltreatment are at an increased risk of revictimization across the life span (Benedini et al., 2016; Finkelhor et al., 2007; Widom et al., 2008). This includes the relationships children have with their peers, which we know play a key role in a range of developmental outcomes (Oshri et al., 2017; Peer, 2006). In other words, the very children who are vulnerable and in need of social support, often experience multiple subsequent adverse social experiences that do not occur at random, but instead disproportionately affect this group (Benedini et al., 2016). Understanding why this happens and how risk for mental health problems unfolds is essential if we are to provide targeted support for those children who are most vulnerable, and perhaps more importantly, develop a preventative model of help that can offset the likelihood of mental health problems emerging.

In the first part of this paper, we systematically consider the evidence for a relationship between childhood maltreatment and peer victimization, and also consider the association with peer rejection, which is more readily observed and investigated in young samples. We then consider the evidence for an association between child maltreatment, peer rejection, and mental health. In particular we are interested to learn if these forms of adverse experience have cumulative or additive effects on mental health outcomes. We then briefly consider a number of theoretical developmental frameworks that have postulated putative mechanisms that might account for the association between child maltreatment and peer victimization. Building on the extant literature, we then consider the role of recent neurocognitive findings as part of a multilevel framework for conceptualizing mental health vulnerability following maltreatment. Neuroimaging research has documented changes in a range of neurocognitive systems, including the threat, reward, and autobiographical memory (ABM) systems (McCrory et al., 2017). We consider how investigation of altered functioning in these neurocognitive systems may shed light on why affected children are at greater risk for a range of deleterious outcomes. Specifically we consider the link between neurocognitive changes and bullying, an example of what can be regarded as “stress generation.” We also discuss how altered neurocognitive functioning may increase the impact of peer victimization (“stress susceptibility”), and at the same time reduce the level of social support that affected children may have to help mitigate the impact of such experiences (“social thinning”).

## Is Child Maltreatment Associated With a Greater Risk of Peer Victimization? A Review of the Evidence

An accumulating body of work has reported a significant association between child maltreatment and peer victimization (Hong et al., 2012; Lereya et al., 2013; Pacheco et al., 2014). Here we comprehensively review the evidence for this association. Child maltreatment is defined as any form of abuse or neglect that occurs to children under 18 years of age (World Health Organization, 2020). Peer victimization is defined as “harm caused by other persons, in this case, peers, acting outside the norms of appropriate conduct” (Finkelhor et al., 2012, p. 3). We prefer to use the terminology “peer victimization” because it has more openness and flexibility than the traditional term bullying as defined by Olweus which requires elements of repeated aggression and power imbalance (Finkelhor et al., 2012; Olweus, 2007). A focus solely on bullying may risk being overly narrow and missing several otinds of negative peer interactions (or patterns of incidental exclusion) that are meaningful in relation to the cycle of violence and their impact on mental health (e.g., Hunter et al., 2007). In addition, we also consider the related concept of peer rejection and its association with child maltreatment. Peer rejection is defined as “the active dislike of an individual on the part of their peers” (Vaillancourt et al., 2013, p. 293) and is more easily observed and investigated in younger sample. Peer rejection has been shown to increase children’s vulnerability to peer victimization (Godleski et al., 2015).

We systematically searched for papers related to child maltreatment and peer victimization and peer rejection. Searches were performed in Google Scholar and PsycINFO, and additional records were retrieved via snowballing. This resulted in approximately 400 records. Following screening of titles and abstracts approximately 300 records were excluded because these records did not focus on the relation between child maltreatment and peer victimization or peer rejection, or sampled adults, psychiatric and/or inpatient populations. Around 100 records were read in full, resulting in 28 papers to be included in this review. The other 72 papers were excluded on the basis of the same criteria, after reading the full-text of each article.

The 28 included papers all addressed the relation between peer victimization/peer rejection and child maltreatment. We included all forms of child maltreatment in this review and separate out those focused on peer victimization in particular (Table 1). Although research has repeatedly shown that there is a relation between peer victimization and peer rejection and that both are related to similar negative outcomes (Vaillancourt et al., 2013) for clarity the two groups of studies will be reviewed separately. Table 1 provides an overview of each study and lists in which country the study was performed, the study design (e.g., longitudinal, cross-sectional), number of participants, type and measure of child maltreatment, peer victimization and peer rejection and key findings.

**Table 1.** Overview of Studies on the Association Between Child Maltreatment and Peer Victimization (Section I) and Peer Rejection (Section II).

Authors	Country	Study Design	Participants	Type and Measure of Child Maltreatment	Type and Measure of peer Victimization/Peer Rejection	Key Findings
Section I: Studies on peer victimization						
Banny et al. (2013)	United States	Summer camp study using cross-sectional data	*N*_maltreated_ = 156, *N*_nonmaltreated_ *=* 145, 37.9% female, *M*_age_ = 11.26, age range 8–13 years	Children in the maltreated group had been identified by the county Department of Human Services (DHS). Classification of maltreatment experiences using the Maltreatment Classification System	Children’s Social Experiences Questionnaire–Self Report (CSEQ-R) to assess peer victimization (overt and relational)	Maltreated children reported significantly higher levels of overt victimization (*p* < .001, *d* = 0.44) and relational victimization (*p* = .001, *d* = 0.37) compared to nonmaltreated children
Benedini et al. (2016)	United States	LONGSCAN, prospective longitudinal study	*N* = 831, 53% female, *M*_age_ = 16.31 years	Physical abuse and sexual abuse. Combination of official reports, child reports, parent reports. Dichotomous measure of maltreatment experience prior to age 12	Juvenile Victimization Questionnaire (JVQ). Reported by participants. Two items: intimidation, and sexual assault	Shows cycle of victimization. Physical and sexual abuse prior to age 12 predicted peer victimization (e.g., intimidation and sexual assault). Association between sexual abuse and intimidation no longer significant when control variables were added
Björkqvist & Österman (2014)	Finland	Cross-sectional design	Study 1: *N* = 1,247, 49% female, grades 7,8,9, *M*_age_= 14.0 years Study 2: *N* = 620, 48% female, grades 7 and 9, *M*_age_ = 13.1 years	Childhood Physical Punishment. Brief Physical Punishment Scale (BPPS). Self-report	Study 1: Victimization. Traditional school bullying was measured with a 28-item scale. Cyber aggression Scale was measured with the 5-item Cyber Aggression Scale (CAS). Self-report. Study 2: Traditional school aggression of three kinds (physical, verbal, indirect) was measured with the Mini-DIA, a short version of the Direct & Indirect Aggression Scales (DIAS)	Study 1: Childhood physical punishment correlated significantly with victimization to traditional bullying in both girls and boys. CPP also correlated with victimization to cyber aggression. Study 2: Victimization to CPP correlates with all measured types of aggression (physical, verbal, indirect)
Bowes et al. (2009)	UK	Environment Risk (E-Risk) Longitudinal Twin Study	*N* = 2,232, age range 5–7 years	Child maltreatment reported by mother	Bullying victimization was measured during interviews with mothers when children were 7 years	Child maltreatment was uniquely associated with an increased risk for being victims of bullying when considered simultaneously with other socioenvironmental factors. Children who experienced maltreatment were approximately twice as likely to be victims of bullying or bully-victims compared with children who had not been maltreated
Calvete et al. (2018) / González-Diéz et al. (2017) (samples from same study)	Spain	Longitudinal study	Calvete et al. (2018): *N* = 1328, 45% female, age range 12–17 years, *M*_age_ = 15.05 years González-Diéz et al. (2017): *N* = 550, 54.5% female, age range 16–19 years, *M*_age_ = 16.97 years	Emotional abuse. Measured with a Spanish adaptation of the Psychological Abuse Scale of the Conflict Tactics Scales Parent-to-Child version (CTS-PC)	Peer Relations Questionnaire for Children (PRQ). Physical and emotional victimization	Calvete et al. (2018): Significant correlations between family emotional abuse at T1 and bullying victimization at T1 (*r* = .171, *p* < .001), T2 (*r* = .154, *p* < .001) and T3 (*r* = .202, *p* < .001) González-Diéz et al. (2017): Significant correlations between Peer victimization T1 and Parents’ emotional abuse at T1 (*r* = .27, *p* < .001)
Chan et al. (2013)	China	Cross-sectional. Two-staged stratified probability sampling procedure in six cities in China	*N* = 18,341, 46.7% female, grades 9–12, *M*_age_ = 15.86 years	Child sexual abuse. Juvenile Victimization Questionnaire, sexual victimization module (12 items)	Child victimization. Juvenile Victimization Questionnaire. Four modules: conventional crime, child maltreatment, peer and sibling victimization, and witnessing of, or indirect, victimization	The findings confirm the association between child abuse and peer victimization
Cluver et al. (2010)	South Africa	Cross-sectional study	*N* = 1,050, 51 female, age range 10–19 years	Participants reported on physical abuse, sexual abuse and domestic violence using seven items from the Child Exposure to Community Violence checklist. Abuse at home is the summed score of physical and sexual abuse	Nine-item standardized Social and Health Assessment Peer Victimization Scale (Ruchkin et al., 2004). Self-report	Abuse at home is a risk factor for bullying victimization, OR_adjusted for covariates_ = 1.34, *p* < .001, 95% CI [1.05, 1.64]. OR_adjusted for all other sign. risk-factors_ = 1.15, *p* < .001, 95% CI [0.85, 1.55]
Hamilton et al. (2016)	United States	Longitudinal study, but cross-sectional relation between PV and CM	*N* = 410, 53% female, age range 12–13 years, *M*_age_ = 12.84 years	Emotional abuse (EA) and emotional neglect (EN) were measured with the EA and EN subscales of the Childhood Trauma Questionnaire (CTQ)	Relationally oriented peer victimization was measured with the Social Experience Questionnaire Self-report (SEQ-S)	Emotional abuse and emotional neglect both were significantly correlated with relationally oriented victimization (*r* = .34 and *r* = .29, respectively)
Holt et al. (2007)	United States	Cross-sectional study	*N* = 689, 48.3% female, age range 10–12 years, *M*_age_ = 10.83 years	Child maltreatment. Juvenile Victimization Questionnaire (JVQ)	Peer victimization. University of Illinois Bullying Scale (UIBS) and Illinois Victimization Scale (UIVS)	Victims reported more child maltreatment than others (*p* < .01)
Hsieh et al. (2016)	Taiwan	Cross-sectional study	*N* = 6233, 49.7% female, fourth graders, *M*_age_ = 10.83 years	Psychological and physical neglect was measured using the 6-item Neglect Subscale of the ISPCAN Child Abuse Screening Tool Children’s Version (ICAST-C)	Bully victimization was measured using a bully victimization scale composed of seven items to measure the respondent’s victimization experience (verbal insult, threats, extortion, property damage, physical violence, and relational aggression).	Significant correlations between bully victimization and psychological neglect (*r* = .29, *p* < .01) and physical neglect (*r* =. 30, *p* <.01)
Hutchinson & Mueller (2008)	United States	Cross-sectional study	*N* = 2,126, 58% female, 6th to 12th grade	Parental emotional abuse. Measured with five items (acts of rejection, condemnation, yelling, nagging, threats of violence, and slapping)	Peer victimization. Five items taken from Kaufman (1999). High score on this index was indicative of an increased level of victimization by types	Parental emotional and verbal abuse increases the odds that a child will become a victim of similar abuse at the hands of his/her peers, both in terms of verbal victimization and physical victimization. Self-esteem as buffer
Lereya et al. (2015)	UK and United States	1. UK Avon Longitudinal Study of Parents and Children (ALSPAC) 2. The USA Great Smoky Mountains Study (GSMS)—longitudinal	ALSPAC: *N* = 4026, 56% female GSMS: *N* = 1420, 49% female	ALSPAC: maltreatment via maternal reports in repeated questionnaire (between ages 1–8 years) GSMS: maltreatment via child and parent report in repeated interviews (between ages 9–16 years)	ALSPAC: Bullied assessed with child reports using the Bullying and Friendship Interview Schedule (at 8,10, and 13 years) GSMS: bullying was assessed with parent and child interviews (part of Child and Adolescent Psychiatric Assessment) (assessed between ages 6 and 9 years)	In both studies, maltreated children were more likely to be bullied than children who were not exposed to maltreatment
Lucas et al. (2016)	Sweden	Nationally representative sample. Cross-sectional	*N =* 3197, 51.5% female, age range 14–15 years	Questions regarding exposure to violence were based on the Conflict tactics Scale. Self-report	Bullying was measured using two questions (have you ever been bullied by other children at school or somewhere else and if you have been bullied, what happened to you?) Self-report	Physical and emotional violence in the home (including experiencing IPV) were significantly associated with bullying victimization. Odds ratios for exposure to bullying rose with increasing frequency and severity of abuse
Meinck et al. (2017)	Romania	Cross-sectional study	*N =* 1733, 55.5% female, 15-year-old students, *M*_age_ = 15.1 years	Physical, emotional, and sexual child abuse victimization were measured using a short version of the Adverse Childhood Experience (ACE) Questionnaire which solely measures abuse, the ACE-ASF	Bullying victimization was assessed using the question: “how often have you been bullied at school in the past couple of months”	Physical/emotional abuse was associated with increased bullying victimization (beta = .215, *p* < .001). Sexual abuse was *not* significantly associated with increased bullying victimization
Radford et al. (2013)	UK	Random UK representative sample	*N =* 2,160 parents and caregivers (of children between 2 months and 10 years), *M*_age_ = 4.6 years *N* = 2,275 children and young people (11–17 years), *M*_age_ = 14.0 years *N =* 1,761 young adults (18–24 years), *M*_age_ = 20.6 years 51.6% female for the entire group	Modified version of the Juvenile Victimization Questionnaire (JVQ)	Modified version of the Juvenile Victimization Questionnaire (JVQ)	Those who had been maltreated by a parent or caregiver in childhood had significantly higher risks of also experiencing victimization by siblings or peers
Schwartz et al. (2000)	United States	Longitudinal study of a representative sample of children; followed for 5 consecutive years (see also Dodge et al., 1994)	*N =* 585, 48.0% female, kindergarten—elementary school	Physical abuse was measured during a 150-min interview with the child’s mother	The peer nomination interview was expanded to include three victimization descriptors (“gets picked on,” “gets teased,” “gets hit or pushed”). Children nominated up to three peers for each item	Significant association between abuse & victimization (.19***). T1 abuse (β = .125, *sr* ^2^ = .012, *p* ≤ .01) were independently predictive of T3 victimization
Shield & Cicchetti (2001)	United States	Summer camp study	*N*_maltreated_ = 169, 35.5% female, *M*_age_ = 8 years, *N*_nonmaltreated_ *=* 98, 35% female, *M*_age_ = 9 years, age range entire group 8–12 years	Parent interview and Child Protective and Preventive Service records. Maltreatment was classified using the Maltreatment Classification System	Bullying victimization. Counselor-report. Mount Hope Family Center Bully-Victim Questionnaire (10 items)	Maltreatment also placed children at risk for victimization by peers. Victims evidenced problems with emotion regulation. In addition, maltreatment’s effects on children’s risk for victimization were mediated by emotion dysregulation
Williamson et al. (2017)	Cambodia	Cross-sectional study	*N =* 206, 50% female, 10th- and 11th grade students at high school, *M*_age_ = 15.1 years	Childhood Trauma Questionnaire short fore; to measure childhood trauma experienced by participants: emotional, sexual, and physical abuse	Peer victimization: measured with a 12-item bullying and victimization assessment	Significant correlations between peer victimization and emotional abuse (*r* = .23, *p* < .01), physical abuse (*r* = .27, *p* < .01), and sexual abuse (*r* = .14, *p* < .05)
Section II: Studies on peer rejection						
Anthonysamy & Zimmer-Gembeck (2007)	Australia	Cross-sectional design	*N* = 400 (25 children with a history of maltreatment), 46% female, age range 4–8 years, *M*_age_ = 6.6 years	Referred by child protection agencies after a notification of abuse by a caregiver	Peer status: sociometric measurement (peer nomination and peer ratings) and teacher reports of peer status. Each teacher nominated three children that other children in the class appeared to most like to play with and three children whom other children in the class appeared to least like to play with. Teachers were also asked to rate how well other children liked to play with each child on a 5-point Likert-type scale	Regardless of the reporter, maltreated children were significantly more disliked, compared with their classmates. Maltreatment had indirect associations with peer likability and peer rejection via maltreated children’s relatively higher levels of physical/verbal aggression and, in some cases, withdrawal and relatively lower prosocial behavior. Maltreatment had an indirect association with teacher-reported peer acceptance via children’s withdrawal
Bolger & Patterson (2001)	United States	Data were drawn from three sources: 1) Charlottesville Longitudinal Study (CLS); 2) Virginia Child Abuse and Neglect Information System (CANIS); 3) Case files in local Departments of Social Services	*N*_maltreated_ = 107, *N*_nonmaltreated_ = 107, 48.6% female, 8–10 years	Information from social service case files; rated according to the Maltreatment classification system	Peer rejection: group sociometric testing. Children were presented with an alphabetized list of children in their grade and were asked to nominate three children whom they liked most and three whom they liked least	Chronically maltreated children are more likely to be rejected by peers across multiple years from childhood to early adolescence. Maltreatment related to aggressive behavior, and this aggressive behavior accounted in large part for association between maltreatment and rejection. Maltreatment chronicity rather than type of maltreatment best predicted rejection by peers
Chapple et al. (2005)	United States	Children of the National Longitudinal Survey of Youth (NLSY-Child). Prospective longitudinal design	*N* = 942, female = 52%, age range 15–17, *M*_age2000_ = 16.0 years	Neglect. Taken from the HOME-SF (mother self-reports or observer reports from maternal behavior)	Peer rejection was measured by two items in which the mothers were asked how true it was that their child “has trouble getting along with other children” and “is not liked by other children”	Child neglect adversely effects peer rejection. Neglected children more likely to be rejected by peers in early adolescence
Chin (2015)	United States	Longitudinal study	*N*_maltreated_ = 100, *N*_nonmaltreated_ = 100, 65% female, age range 9–12 years, *M*_age-maltreated_ = 10.54 years, *M*_age-nonmaltreated_ = 10.50 years	Confirmed cases of physical abuse, coded from CPS records	Peer rejection; peer nomination assessment—sociometric negative nominations by classmates. Only at T1	Mean peer rejection differs (*d* = 0.6) between abused (*M* = .39, *SD* = .49) and control (*M* = .13, *SD* = .34). Physical abuse at T0 was significantly associated with Peer rejection at T1: OR = 4.28, *p* < .001, 95% CI = 2.11-8.69
Dodge et al. (1994)	United States	Longitudinal study of a representative sample of children; followed for 5 consecutive years	*N* = 585 (12% identified as having experienced physical maltreatment), 48% female, kindergarten—elementary school	Physical maltreatment: clinical interview with parents	Peer relations. Peer sociometric interviews: each child was shown a roster of all classmates and was asked to rate how much he or she liked that classmate on a 5-point scale. Next, the child was asked to nominate up to three peers as especially liked and disliked.	Peer, teachers, and mothers independently evaluated the maltreated group of children as being more disliked, less popular, and more socially withdrawn than the non-maltreated group in every year of evaluation, with the magnitude of difference growing over time.
Downey & Walker (1989)	United States	Cross-sectional study	*N_maltreated_* = 25, 60% female, *M*_age_ = 8.9y; *N*_nonmaltreated_ = 28, 39% female, *M*_age_ = 9.9y	Families with currently active CPS files were invited to participate. Abuse charges were legally substantiated for only 10-15%.	Peer rejection. Based on the CBCL (mother-reported). Peer rejection scale (3 items)	Correlation between maltreatment and peer rejection is .23 (*p* < .05). Maltreatment was positively associated with peer rejection, controlling for age and psychiatric status (see table 3)
Fantuzzo et al. (1998)	United States	Cross-sectional study	*N*_maltreated_ = 54, *N*_nonmaltreated_ = 54, 57.4% female, age range 2.9–4.8 years, *M*_age_ = 3.9 years	Maltreatment. Substantiated records of maltreatment	Sociometric ratings. Peer ratings were obtained by asking children to assign classmates photos to bowls varying in size to indicate degree of preference: “like to play with,” “do not like to play with”	Maltreated children were significantly more likely than their non-maltreated peers to receive low sociometric ratings and significantly less likely than non-maltreated peers to receive highs sociometric ratings from their peers
J. Kim & Cicchetti (2010)	United States	Summer camp study	*N*_maltreated_ *=* 215, *N*_nonmaltreated_ = 206, 34% female, age range 6–12 years, *M*_age_ = 8.11 years	Maltreatment. Identified through the County Department of Social Services (DSS). Specific maltreatment experiences were coded with the Maltreatment Classification System (MCS)	Peer rejection. Measured with peer nomination: name a peer from the group whom they liked most, liked least, and who best fit behavioral descriptions (e.g., cooperative, disruptive)	Maltreated children had higher levels of peer rejection, and also had lower levels of peer acceptance with a marginal significance. This study also showed the important role of emotion regulation as a risk or a protective mechanism in the link between child maltreatment and later psychopathology through its influence on peer relations
Levendosky et al. (1995)	United States	Cross-sectional study	*N* = 68, female = 51.5%, *M*_age_ = 10.27 years	Child Protective Services records were checked to verify the presence or absence of any substantiated maltreatment	Parents and teachers completed the CBCL. A peer rejection subscale was created consisting of three items	Maltreatment was *not* significantly related to peer rejection (*r* = .17*, p* > .05)
Salzinger et al. (2001)	United States	Cross-sectional study	*N*_physically-abused_ = 100, 35% female, age range 9–12 years, *M*_age girls_ = 10.6 years, *M*_age boys_ = 10.5 years *N*_nonabused classmates_ = 100, 35% female, age range 9–12 years	Physical abuse. Reported in the NY State Register for Child Abuse. Sexually abused children were sexually excluded, but children who were neglected and physically abused were not	Peer rejection. Sociometric ratings. Peer rejection is based on both the positive and negative two-choice procedure is based in on algorithm in which children are classified as rejected if they receive only negative choices	Significant correlations between agency-confirmed abuse (.30***), abuse index (.19***) and peer rejection. Nonsignificant correlation between abuse severity index (.12) and peer rejection

In total, 18 studies reported on the association between child maltreatment and peer victimization (see Table 1, Section I). In each of the studies reviewed, a significant association between child maltreatment and peer victimization was reported. This association was observed, irrespective of whether studies focused on maltreatment generally, or on individual subtypes of maltreatment (however, see Meinck et al., 2017, in relation to sexual abuse). The association was also observed irrespective of gender (e.g., Calvete et al., 2018; Schwartz et al., 2000). Although most studies focused on children between 10 and 16 years, child maltreatment and peer victimization was evident in children in different age groups, from children in kindergarten and elementary school (e.g., Banny et al., 2013; Schwartz et al., 2000), to children in high school (e.g., Calvete et al., 2018), and those in late adolescence (Radford et al., 2013). Although age was frequently controlled for in the analyses, there was one study that showed evidence that the relationship between child maltreatment and peer victimization became stronger over time (Radford et al., 2013). There was also some evidence that the likelihood of peer victimization was greater when the frequency and severity of abuse increased (Lucas et al., 2016).

We found 10 studies that reported on the association between child maltreatment and the related construct of peer rejection (see Table 1, Section II). Peer rejection is often captured by a sociometric measurement based on peer nominations (e.g., Bolger & Patterson, 2001; Salzinger et al., 2001). All but one study (Levendosky et al., 1995) found a significant association between child maltreatment and peer rejection. Again, this was found irrespective of whether studies conceptualized maltreatment as a broad construct, or focused on individual maltreatment subtypes (however, see J. Kim & Cicchetti, 2010 in relation to neglect). As with peer victimization, effects were comparable across boys and girls (Bolger & Patterson, 2001; Dodge et al., 1994; Downey & Walker, 1989; Fantuzzo et al., 1998; Salzinger et al., 2001). It was striking that evidence of an increased likelihood of peer rejection was evident even from infancy (e.g., Fantuzzo et al., 1998). However, as with peer victimization, there was one study that showed that the association between child maltreatment and peer rejection strengthened over time (Dodge et al., 1994).

In light of this extant evidence, there appears to be a robust relationship between child maltreatment and peer rejection and peer victimization. Overall, maltreated children are typically two to four times more likely to be victimized or rejected by their peers than nonmaltreated children. Although age was frequently controlled for in the analyses, there is tentative and preliminary evidence from two studies the relationship between child maltreatment and peer victimization may become stronger over time (Dodge et al., 1994; Radford et al., 2013). This requires further investigation. The studies covered in this review vary in their study designs: while the majority were cross-sectional in nature, a number adopted a longitudinal design (Benedini et al., 2016; Dodge et al., 1994; J. Kim & Cicchetti, 2010; Lereya et al., 2015; see Table 1). Such designs are necessary if we are to gather evidence for a causal association between maltreatment experience and victimization, often referred to the “cycle of victimization,” which postulates that child maltreatment precedes social rejection (Widom, 2014). The inference that peer victimization follows on from prior maltreatment experience is most strongly supported from the findings of longitudinal studies (Benedini et al., 2016; Dodge et al., 1994; J. Kim & Cicchetti, 2010; Lereya et al., 2015), although more longitudinal data using causal inference methods (e.g. genetically informative or propensity score matching designs) are needed. The longitudinal studies consistently found that maltreatment accounted for increased probability of subsequent victimization or rejection. This presents a compelling question: *how* does maltreatment act in a way to potentiate the risk of later victimization? Later on, in this paper, we discuss several theoretical frameworks that have sought to shed light on this association. Before we do so, we first discuss the impact of child maltreatment and peer victimization on mental health.

## What Is the Impact of Child Maltreatment and Peer Victimization on Mental Health?

Child maltreatment has serious detrimental and long-lasting effects on children’s mental health (Hillberg et al., 2011; Leeb et al., 2011; Oswald et al., 2009). For example, child maltreatment has been associated with anxiety, aggression, depression, and substance abuse disorders (Johnson et al., 2002; Scott et al., 2010). Research has also shown that peer victimization is significantly associated with negative mental health outcomes including, for example, externalizing problems, psychosomatic problems, psychotic symptoms, self-harm, and suicide (Arseneault et al., 2010; Gini & Pozzoli, 2013; Moore et al., 2017; Reijntjes et al., 2011; Ttofi et al., 2011; Van Geel et al., 2015). Longitudinal studies indicate that this association holds even when baseline mental health problems and genetic confounds are accounted for in the analyses (Arseneault et al., 2006; Bond et al., 2001; Y. S. Kim et al., 2006; Singham et al., 2017). Peer rejection has also been significantly associated with negative mental health outcomes, while peer acceptance is associated with improved mental health outcomes (e.g., Coie et al., 1992; Ladd, 2006; Ladd & Troop-Gordon, 2003; Pedersen et al., 2007; Platt et al., 2013; Reisman, 1985; Ueno, 2005).

Only a small number of studies have systematically investigated child maltreatment *alongside* peer victimization, which is required to gain a better understanding of how these experiences may reciprocally influence each other over time. Extant longitudinal studies of young adolescents that have controlled for potential confounding variables have found independent effects of childhood maltreatment and peer victimization on psychotic symptoms (Arseneault et al., 2011), depression (Hamilton et al., 2013), and self-harm (Fisher et al., 2012). Such effects were found even when genetic risk was controlled (Arseneault et al., 2011). Studies of older adolescents and young adults show a similar pattern with regard to mental health outcomes. In contrast to the studies on young adolescents, these studies were cross-sectional in design. Gren-Landell et al. (2011) reported that both lifetime maltreatment and peer/sibling victimization were related to social anxiety disorders in female but not male adolescents. Gibb et al. (2004) found that verbal victimization conferred cognitive vulnerability to depression in young adults over and above the effects of emotional maltreatment. Duncan (1999) reported that first year college students who had been exposed to maltreatment and peer victimization (i.e., bullying) had higher levels of PTSD than those who had experienced maltreatment only.

Collectively, these findings indicate that maltreatment and peer victimization have significant and unique effects on children’s mental health. However, it is important to acknowledge the interdependency that likely exists between child maltreatment, peer victimization, and poor mental health. An increase in mental health problems (e.g., internalizing or externalizing behaviors) following maltreatment experience may well increase the risk of peer victimization. This experience would then likely exacerbate mental health problems further. Evidence for such a pathway requires longitudinal studies that can take into account baseline levels of mental health problems and experiences of peer victimization. For example, Kim and Cicchetti (2010) showed that higher externalizing symptomatology (Time 1) contributed to later peer rejection (Time 2) which in turn was related to higher externalizing symptomatology (Time 2). Evidence of such iterative effects underlines the importance of delineating how vulnerability *unfolds over time* rather than considering the impact of these stressor events as discrete and independent. Later we present a neurocognitively informed model that seeks to address this issue.

Recent findings from genetically informed studies provide further evidence of the interdependency between child maltreatment, peer victimization, and poor mental health. In a longitudinal study investigating schizophrenia Riglin and colleagues (2018) found that while the early development of emotional problems was associated with genetic risk, the subsequent course of emotional problems was potentiated by exposure to future peer victimization. Genetically informative studies have also shown that genetic factors that increase vulnerability to mental health problems also increase vulnerability to peer victimization (Schoeler et al., 2019). Schoeler and colleagues (2019) demonstrated that polygenic risk scores for depression and attention-deficit/hyperactivity disorder denoted increased risk of peer victimization, suggesting that preexisting genetically driven mental health vulnerabilities are risk factors for exposure to peer victimization.

## Why Does Child Maltreatment Increase the Risk of Peer Victimization?

The relationship between child maltreatment and peer victimization has been studied and understood within a broad range of different theoretical frameworks. Bowlby’s attachment theory (1969) proposes that maltreated children are less likely to form secure attachment relationships with their primary caregivers. As a consequence, these children develop a distorted internal working model which in turn puts them at risk for the development of deviant peer relationships. Research has indeed shown that child maltreatment is related to insecure attachment relationships (for meta-analyses see Baer & Martinez, 2006 and Cyr et al., 2010) and that lower attachment security seems to be carried forward into relationships with peers (Morton & Browne, 1998; Wood et al., 2004; Youngblade & Belsky, 1989). Finkelhor’s developmental victimology framework significantly extended this work, by highlighting the role played by environmental risk factors on the one hand (for example parental behavior or family instability) and personal characteristics of the child on the other (Finkelhor & Asdigian, 1996).

These approaches are encompassed by the ecological–transactional model of child maltreatment, a highly influential framework put forward by Cicchetti and colleagues (Cicchetti et al., 2000). According to this model, multiple levels of a child’s ecology influence each other and in turn also influence a child’s development (Belsky, 1993; Bronfenbrenner, 1979; Cicchetti & Lynch, 1993; Sameroff, 2009). Moreover, according to Cicchetti’s organizational perspective (2016), children’s development is considered as a hierarchical process in which the successful completion of earlier stages increases the likelihood of subsequent successful adaptation (Cicchetti, 1993, 2016; Sroufe, 2013). Developmental tasks include, for example, deploying effective emotion regulation and formation of functioning social relationships. Child maltreatment is thought to hinder successful attainment of these developmental goals (e.g., Benedini et al., 2016), which may then hamper the individual’s capacity to achieve subsequent developmental milestones. For example, inability to appropriately regulate emotions has consequences for negotiating complex social relationships both within and outside the family, increasing risk of peer rejection and peer victimization (Cicchetti, 2016; J. Kim & Cicchetti, 2010).

The model also encompasses social learning as mechanism of vulnerability to peer rejection following maltreatment. In families where maltreatment occurs, children witness aggressive and coercive behavior by adults and observe that it can result in compliance by others. A child exposed to maltreatment may then imitate this coercive style during their interactions with peers. Many studies have shown that aggressive and coercive behavior by a child is associated with increased risk of rejection by their peer group (see for a meta-analysis Newcomb et al., 1993). In line with this social learning framework, Downey et al. (1994) have postulated that rejection sensitivity plays a key role in victimization (Downey et al., 1994; Levy et al., 2001). Specifically, rejection by maltreating parents might engender in the child the expectation of rejection by others. Anxious expectations of rejection may then foster a hypervigilance for rejection; as such, even though the child’s perception of rejection may be inaccurate, their negative responses to other’s ambiguous behaviors may produce an actual rejection experience and a self-fulfilling prophecy (Downey et al., 1994).

The ecological–transactional model explicitly frames these factors within a multilevel context, acknowledging the interplay of biological, psychological, and environmental risk factors across development (Cicchetti et al., 2000). However, although the field has made significant progress in documenting neurobiological correlates of maltreatment in general (Cicchetti & Toth, 2005; McCrory et al., 2017) there has been relatively little focus on how such changes in the brain specifically relate to peer victimization. There is a need to build on this work by providing greater specificity at a neurocognitive level in relation to how maltreatment experience shapes information processing and how this in turn influences social interactions across development. By delineating more precisely these developmental mechanisms we will be better placed to inform approaches to prevention and intervention.

## Advances in Neurocognitive Research: What Can It Tell Us About the Cycle of Vulnerability?

In recent years neuroimaging research has begun to document alterations in a range of neurocognitive systems, including threat, reward and ABM processing as well as emotion regulation in children who have experienced maltreatment (for detailed reviews of neuroimaging findings in the field of maltreatment please see McCrory et al., 2017 and McLaughlin et al., 2019). The theory of latent vulnerability has postulated that these alterations can be understood, at least in part, as adaptations to early adverse or neglectful early environments in line with the notion of experiential canalization (McCrory et al., 2017; McCrory & Viding, 2015). While such adaptations are thought to provide short term functional advantages in atypical early environments, they are believed to contribute to mental health vulnerability over the longer term. It has been proposed that such vulnerability is likely to emerge both as a result of atypical processing of internally and externally generated experience as well as through a socially mediated pathway (McCrory et al., 2019). Below we discuss how neurocognitive alterations in three systems may increase both the *likelihood* of bullying as well as the *impact* of bullying and consider the implications of this for mental health vulnerability.

## Can Neurocognitive Research Help Shed Light on *Why* Children Are More at Risk of Peer Victimization Following Maltreatment?

Alterations in the threat system of children who have experienced maltreatment have perhaps been the most widely documented (Miller, 2015). Relative to matched peers these children show increased activation of regions implicated in salience processing, including both the amygdala and anterior insula (McCrory et al., 2011; Teicher et al., 2016). For example, we have reported that children exposed to documented maltreatment, during incidental processing of angry versus neutral adult facial expressions in a gender decision task, show increased activation in the amygdala and anterior insula compared to peers with no maltreatment experience (McCrory et al., 2011). This pattern of altered threat processing has also been observed even at a preconscious stage of awareness. Using a dot probe paradigm, with facial cues presented preattentively (i.e., for 17 ms) and backward masked, children exposed to documented maltreatment compared to peers showed greater neural response in the amygdala to angry relative to neutral faces, suggesting that neural response to threat cues in maltreated children is not the result of conscious regulatory control. Hypoactivation of this same system has also been observed alongside a pattern of behavioral avoidance of threat cues during a social rejection-themed emotional Stroop task; this neural and behavioral profile has been associated with symptoms of dissociation (Puetz et al., 2016). These findings have been postulated to reflect a complex pattern of adaptation to threat that may be helpful for a child managing in a chaotic or dangerous home environment, increasing their capacity to both rapidly identify new sources of threat as well as manage overwhelming feelings of anxiety and vulnerability (McCrory & Viding, 2015). However, in more normative environments these adaptations may result in both: (i) processing of negative cues that inappropriately prime either a conflictual response or an avoidant / dissociative response (what has commonly been understood as a “fight or flight” reaction) as well as (ii) increase stress reactivity—that is, potentiate or magnify the signal generated during new stressor experiences that places an ongoing burden on the individual’s emotion regulation system. It is not difficult to imagine how such patterns of response in the context of unsympathetic peers, may increase the possibility of peer rejection or even victimization.

In recent years, researchers have extended their focus to investigate functioning of the reward system in children who have experienced abuse and neglect. The reward system has been implicated in incentive-based learning and goal-directed behaviors, which are critical for decision-making and cultivating and maintaining social relationships. In typical early environments the caregiver provides predictable reinforcements that include food, pleasant, soothing touch, and signals of positive and negative affect. Therefore, during early development, each individual builds a model of the predictability and structure of the world that is calibrated by the caregiver’s behavior. This model in turn is associated with that individual’s ability to successfully make accurate predictions when navigating new social environments. Several studies have documented that maltreatment experience is associated with attenuated neural response in key reward processing areas of the brain, during anticipation and consummation of rewards, or when learning or relearning the reward value of a stimulus (e.g., Dillon et al., 2009; Gerin et al., 2017; Goff et al., 2013; Hanson et al., 2015; Mehta et al., 2010). For example, in a large community sample of adolescents with varying degrees of childhood maltreatment Hanson and colleagues (2015) found that severity of emotional neglect was associated with reduced development of striatal neural response during receipt of monetary rewards. This blunted neural response was found to partially mediate the association between a history of neglect and depressive symptomatology 2-year postbaseline.

These initial findings are perhaps unsurprising, when we consider how the reward system might be shaped by experiences of neglect and abuse. In a home characterized by neglect, rewards are infrequent and unpredictable. In abusive environments, threat and reward may both be unpredictable and may not be reliably associated with cues (and close relationships) that typically signal reward or punishment. In other words, maltreatment exposure may lead to adaptation of the reward system, such that it is poorly optimized to make predictions or evaluate reward value of stimuli in a way that assists adaptive decision-making and social interactions in more typical environments. This is highly relevant in the context of social rejection. If children are less motivated and less able to engage appropriately with others, perhaps in part because they interpret social cues inaccurately, they will respond to others in ways that appear unpredictable and “off-the-mark.” Over time, unpredictable, socially inappropriate or withdrawn behavior may potentiate rejection or even victimization by peers and adults.

Alterations in the ABM system may also be pertinent when considering vulnerability to social rejection in individuals who have experienced abuse and neglect. ABM refers to retrieval of personally experienced events. The accurate and successful retrieval of autobiographical events is thought to help scaffold our sense of self, develop and maintain social bonds, and plan new actions (Alea & Bluck, 2003; Conway & Pleydell-Pearce, 2000; Nelson, 1993; Pillemer, 2003; Williams et al., 1996). ABM is so critical because it facilitates our ability to use past experiences to construct representations of others, their mental states, and actions. It has been shown that children who have experienced maltreatment show a pattern of over-general ABM characterized by a less detailed recollection of personal experiences (Valentino et al., 2009). For children who have had traumatic experiences, less vivid and detailed memories may be adaptive in serving to minimize negative affect but this processing style becomes problematic when it is generalized to everyday memories. Overgeneral memories are thought to limit self-projection in future scenarios and impede social functioning by limiting social problem-solving skills. At the neural level, ABM in the context of maltreatment has been investigated using a standard task where neural response is measured during the recall of well specified positive and negative memories that have been generated by participants prior to the scanning session (McCrory, Puetz, et al., 2017). Under such conditions, maltreatment experience is associated with altered brain responses during recall of negatively and positively valenced memories: negative memory recall elicits greater activation of the salience network including the amygdala, whereas positive memory recall elicits reduced activation of the hippocampus (McCrory, Puetz, et al., 2017). In this light, altered ABM has been postulated to be associated with increased risk of peer rejection and peer victimization by compromising social problem solving and increasing focus on the negative, while reducing processing of positive social information.

It is striking that the neurocognitive alterations associated with the threat, reward and ABM systems following maltreatment experience are similar to what is observed in individuals presenting with manifest clinical disorders including anxiety, depression, and conduct disorder. Moreover, childhood maltreatment, as a powerful stressor experienced during potentially critical period of development, may lead to functional and structural neurological alterations, which appear to be linked to increased generalized vulnerability to adverse mental health outcomes. Exaggerated behavioral response to threat and heightened neural activation to threat are seen in individuals with anxiety disorder for example, as well as a subset of children with conduct disorder (Gerin et al., 2019). Attenuated neural activation of the reward system is seen in individuals at risk of developing or with current depression. A pattern of overgeneral memory recall, as well as heightened neural activation of the salience network during negative ABM recall is observed in individuals at risk of developing or with current depression. Recent genetically informative work has found that polygenic risk indexing mental health vulnerability, including depression, is associated with an increased risk of being bullied. That is, it appears that bullying (and therefore social rejection) does not happen at random, but may in part be accounted for by genetic factors (i.e., gene–environment correlation) (Ball et al., 2008; Schoeler et al., 2019). In the same vein, neurocognitive adaptation following maltreatment experience may lead to information processing patterns that increase mental health vulnerability that in turn increase risk of peer victimization (which can be considered a form of environment–environment correlation). In this way a cycle of vulnerability may be set up, whereby neurocognitive adaptation following childhood maltreatment increases risk of peer victimization directly, but also indirectly via development of mental health problems.

## Conclusion

Children who experience maltreatment are at increased risk of being rejected and victimized by their peers. Not only will this negatively impact their wellbeing, we have good evidence to believe that both child maltreatment and peer victimization will independently contribute to poorer mental health outcomes. To date child maltreatment and peer victimization have been studied within a broad range of theoretical frameworks, including attachment theory, the ecological–transactional model of child maltreatment, the social-learning framework, and the developmental victimology framework. What is striking is the absence of neurocognitive specificity within these models in explaining the association between child maltreatment and peer victimization and its relation with mental health.

The neurocognitive findings that we have reviewed suggest that alterations in brain function following maltreatment experience may be important to consider when we seek to understand the relationship between maltreatment and peer victimization. It has been suggested that early adversity in the form of maltreatment may lead to brain adaptations that may be adaptive in an early atypical home environment, but which can derail social functioning with peers, thereby increasing the likelihood of peer rejection or victimization. Such exposure to new stressors is then likely to contribute to an increased risk of developing mental health problems or exacerbating existing problems.

We have previously conceptualized three pathways that capture how such mental health vulnerability can unfold following maltreatment. Here we argue that peer victimization can be considered as an example of one of these pathways: stress generation. We have suggested that altered neurocognitive functioning can mean that stressful experiences are more likely to occur for individuals who have experienced maltreatment in childhood (Gerin et al., 2019). An individual who shows an exaggerated response to real or perceived threat (e.g., withdrawal or aggression), who does not respond typically to social and instrumental rewards and who may not excel at social problem solving will be more likely to behave in ways that precipitates new stressful social interactions. This can include an increased likelihood of peer victimization. To date, neuroimaging studies have not systematically investigated the relationship between altered neurocognitive functioning following maltreatment experience and stress generation. One community study of adolescent girls has found a relationship between neural response to reward and stress generation, suggesting that altered reward processing may be an appropriate target for future investigation (Mackin et al., 2019). A child may also be more vulnerable to peer victimization if they have a weaker or attenuated social support network. We have suggested that altered neurocognitive functioning can mean social bonds are less likely to be successfully cultivated or maintained over time. This second pathway has been termed social thinning (McCrory, 2020). An individual who behaves in ways that disrupt harmonious and predictable social interactions is less likely to elicit support and induce others to expend effort in maintaining close social bonds with them. Over time, this reduces the quality and number of trusted social relationships that can help buffer experiences of future stress, as well as reduce the likelihood of peer victimization (Kendrick et al., 2012; Ttofi et al., 2011). Given that amygdala connectivity is known to predict social network size in adults, and the relationship between conduct disorder (which is associated with heightened neural response to threat cues) and poor social outcomes in adulthood, it may be pertinent to investigate how altered threat processing contributes to social network development in children with maltreatment experience.

In relation to mental health vulnerability, we and others have argued that altered neurocognitive functioning can mean that everyday life extracts a greater toll (Danese & McEwen, 2012; Gerin et al., 2019). We call this third pathway stress susceptibility. At the neurocognitive level this can be understood as a constellation of: (i) amplified reactivity to threat (real or perceived); (ii) attenuated responsivity to rewarding stimuli; and (iii) reduced social competence, characterized by limited behavioral repertoires that can mitigate the impact of stressful experiences. In sum, neurocognitive alterations that follow early adversity mean that peer rejection and peer victimization is more likely to occur (stress generation), especially in a context where a child may be less able to cultivate and maintain the social support of peers (social thinning). When such victimization occurs, the effect may be amplified (stress susceptibility).

We need to conduct novel, systematic research in order to develop a preventative model of help, targeted to support those children who are most vulnerable to being revictimized. Such a preventative model of help is required to offset the likelihood of mental health problems emerging. Ideally, this research will be longitudinal and will include sensitive measures both of social functioning as well as of discrete neurocognitive processes altered following maltreatment experience and implicated in social behavior. It will be important to delineate with more precision the way in which social behaviors are influenced by altered neurocognitive functioning that compromise optimal interaction with peers. In other words, there needs to be a more explicit characterization of how prior maltreatment experience and genetic vulnerability alter neurocognitive functioning in ways that meaningfully shape everyday social behavior, and relate such changes in turn to a child’s ability to cultivate and maintain social relationships with peers. Such empirical research is necessary to inform any theoretical model of how altered neurocognitive functioning following maltreatment experience may be related to victimization. From a methodological perspective, this would require identifying children with carefully documented maltreatment experience at different points of development and following them up longitudinally using a combination of experimental, neuroimaging, social and mental health/well-being measures. The challenge for the field is to improve the psychometric properties of experimental/neuroimaging probes and to validate these so that the same constructs can be reliably assessed at different developmental stages. Furthermore, measurement of social relationships needs to be innovative (combination of rating scales, social network measures and utilizing ecological momentary assessment technologies) and chart the relationships most pertinent to the child’s current developmental stage.

In order to examine these and other questions we need designs and measures that have sufficient sensitivity to capture individual differences and that study child maltreatment alongside social functioning and mental health. Individual differences can for example be captured by intensive longitudinal methods such as experience sampling method (Larson & Csikszentmihalyi, 2014) or ecological momentary assessment (EMA; Shiffman et al., 2008). EMA can also include physiological measurements which may be useful in examining the stress susceptibility model. Using intensive longitudinal methodologies allows the study of the dynamics of social relationships and mental health over time. Another area worth exploring is experimental manipulation of social situations, for example by using virtual reality or microtrial designs (Howe et al., 2010; Parsons, 2015). These may create opportunities to recalibrate specific neurocognitive systems, particularly threat and reward processing, that may be implicated in vulnerability to victimization. These insights could be implemented in tools for (preventative) interventions for children and adolescents (Nocentini et al., 2015) with the aim of reducing the likelihood of children who have been exposed to maltreatment experiencing new forms of stress through peer victimization.

### Implications of the Review for Practice, Policy, and Research

#### Practice

- Caregivers and teachers should pay particular attention to the social development of children with documented or suspected maltreatment / neglect experience. Particular attention should be paid to early signs of peer victimization / rejection.
- Clinical and social work professionals should ensure that assessments explicitly cover social functioning when mapping risk and vulnerability factors, and include actions / recommendations to support social functioning in any treatment or care plan.
- Consider creating buddy systems (or similar strategies) for at risk children with maltreatment experience to help them cultivate and maintain social relationships and strengthen their social support network.
- Explicitly promote social skills development in at risk children, drawing from evidence-based programs focusing on social competencies (emotion regulation etc.).
- Clinical interventions should include a focus on social information processing which help children to detect and interpret affective cues accurately and generate appropriate behavioral responses.

#### Policy

- Anti-bullying programs should incorporate maltreatment experience as a risk factor for peer victimization and peer rejection.
- Child maltreatment and peer victimization have significant and unique effects on children’s mental health. That is, both have a negative impact and require distinct responses.
- Research suggests that neurocognitive alterations may contribute to an increased likelihood of peer relationship difficulties; however, the brain remains malleable across childhood, and continues to be responsive to new experiences, particularly in the context of trusted relationships.
- It is important to consider preventative intervention approaches *before* mental health problems emerge. These have the potential to reduce the impact and cost of later problems.

#### Research

- More longitudinal studies are needed to gather evidence for a causal association between child maltreatment and victimization, and that take into account baseline levels of mental health problems and experiences of peer victimization.
- To gain a better understanding of how risk factors operate in relation to mental health problems and how vulnerability unfolds over time, we need studies that systematically investigate child maltreatment alongside peer victimization.
- Research is needed to provide greater specificity at a neurocognitive level in relation to how maltreatment experience shapes information processing and how in turn influences social interactions across development.
- There needs to be a more explicit characterization of how prior maltreatment experience and genetic vulnerability alter neurocognitive functioning in ways that meaningfully shape everyday social behavior, and relate such changes in turn to a child’s ability to cultivate and maintain social relationships with peers.
